# Advanced hybrid nanomaterials

**DOI:** 10.3762/bjnano.10.247

**Published:** 2019-12-20

**Authors:** Andreas Taubert, Fabrice Leroux, Pierre Rabu, Verónica de Zea Bermudez

**Affiliations:** 1 Institute of Chemistry, University of Potsdam, Karl-Liebknecht-Str. 24–25, D-14476 Potsdam OT Golm, Germany; 2 Chemical Institute of Clermont-Ferrand, UMR CNRS 6296, University Clermont Auvergne, 24 av. Blaise Pascal, Aubière, France; 3 Institute of Physics and Chemistry of Materials of Strasbourg, CNRS-University of Strasbourg, 23, rue du Loess, BP43, Strasbourg cedex 2, France; 4 Departamento de Química - Escola de Ciências da Vida e do Ambiente, Universidade de Trás-os-Montes e Alto Douro, 5001-801 Vila Real, Portugal

**Keywords:** colloidal chemistry, environmental remediation, hybrid nanomaterials, nanocomposite, nanofillers, nanomedicine, nanostructures, polymer fillers, pore templating, smart materials

The Maya blue pigment that was used in Mexico during the VIIIth century is often given as a prototypical example of a hybrid material in which an indigo derivative is stabilized into magnesium aluminum phyllosilicate. This material, palygorskite, was used to produce beautiful mural paintings that are still shining centuries later [1–3]. Since these origins, the field of hybrid materials has developed into a broad scientific and technological subject including important fields such as sol–gel chemistry [4–5], polymer nanocomposites [6–7], and hybrid nanomaterials [8–9]. Nowadays, hybrid materials are almost everywhere and provide a wide range of applications from biology and health, to photonic devices, catalysis and environment, smart coatings, energy, and electronics [10–17]. By essence, the hybrid approach consists of building new materials and devices by assembling elementary functional organic/inorganic, molecular or extended bricks to obtain materials with greatly improved or even completely new properties. This has been called a Lego^©^-like approach to materials synthesis [18]. The main issue in this approach is to monitor and control interactions between different bricks and to understand the physico-chemical mechanisms involved at the interfaces between the individual building blocks as well as between different materials. Moreover, the implementation of hybrid systems in devices implies miniaturization. Therefore, nanostructuration or nanoarchitectonics is the core of current research in hybrid materials, and the analysis of advanced hybrid materials needs specialized experimental and theoretical techniques.

This thematic issue clearly shows that “advanced hybrid nanomaterials” is not just hype, it is a real and powerful toolbox towards advanced materials for highly diverse fields, such as polymer nanocomposite, health and environment.

## Smart Materials and Nanostructures

As far as hybrid materials are concerned, significant effort is still focused on materials synthesis and characterization. This is most probably due to the endless number of combinations of different moieties that can be envisaged, as well as to the increasingly complex, resulting structures with critical importance of interfaces. In the current issue, from “simple” to more elaborated, we here observe the importance of the polyol method, the non-hydrolytic or colloidal approach, and ordered mesopore templating techniques.

In “Tailoring the magnetic properties of cobalt ferrite nanoparticles using the polyol process” [19], the synthetic process was found to be of prime importance to shape the nanoparticles and to optimize their surface/volume ratio in relation to the magnetic behavior.

A one-step non-hydrolytic sol–gel synthesis of mesoporous TiO_2_ phosphonate hybrid materials was applied to yield diverse materials, which were found to depend on the P/Ti atom ratio [20]. The ratio was found to determine the particle size and the aggregation state and thereby could strongly tune the porosity of the resulting materials.

Colloidal chemistry with patchy silica nanoparticles was employed to synthesize clusters, so-called colloidal molecules [21]. Nanospherical satellites were covalently bonded via amide groups within the dimples of valence-endowed patchy nanoparticles, allowing the tuning of their topology and self-assembling ability.

Polyion complex micelles formed by complexation between poly(ethylene oxide)-*b*-poly(acrylic acid) (PEO-*b*-PAA) and an oligo-chitosan-type polyamine was used as a structure-directing agent to prepare ordered mesoporous silica materials in the work “pH-mediated control over the mesostructure of ordered mesoporous materials templated by polyion complex micelles” [22]. The mesostructures are highly pH-sensitive, adopting 2D-hexagonal, wormlike or lamellar organization depending on the extent of the electrostatic complexing bonds and on the condensation rate.

More complex assemblies involving ternary compositions in “Ternary nanocomposites of reduced graphene oxide, polyaniline and hexaniobate: hierarchical architecture and high polaron formation” were explored for the promotion of synergistic effects expected at the nanoscale [23]. The resulting mixture between polyaniline chains with reduced graphene flakes and hexaniobate nanoscrolls may find application as coatings for sensing or corrosion protection.

To understand composite formation of a complex hybrid assembly, high quality characterization is paramount. An example is small angle X-ray scattering (SAXS), which was used in the work “Mechanism of silica–lysozyme composite formation unraveled by in situ fast SAXS” to identify and characterize subtle interparticle interactions [24]. This study shows that fast in situ synchrotron SAXS provides an understanding of lysozyme deformation molecules during aggregation. All these contributions indicate a marked interest of current research in hybrid materials for nanostructuration and related issues.

## Nanofillers

Nanocomposites remain a vast playground for research into new hybrid systems. A common approach for polymer fillers presented in this thematic issue is their use as organo-modified layered double hydroxides (LDHs) or new layered calcium phenylphosphonates, as well as functionalized films with high dielectric constant, or, in the case of optical applications, this consists also in embedding different types of nanoparticles.

In the first example, “Co-intercalated layered double hydroxides as thermal and photo-oxidation stabilizers for polypropylene”, the concomitant intercalation of both a thermal and a photo-oxidation stabilizer endows polypropylene with remarkable resistance against thermal degradation and photo-oxidation [25]. In the same vein, the protection of the polymer using organo-modified LDH was addressed in “Outstanding chain-extension effect and high UV resistance of polybutylene succinate containing amino-acid-modified layered double hydroxides” [26]. However, this time with a “green aspect”, since the polymer studied is a bio-based polyester and the organo-modifying agent of the 2D-filler is an amino acid.

The same host structure (i.e., LDH) was employed in “Topochemical engineering of composite hybrid fibers using layered double hydroxides and abietic acid” [27]. In this work, a composite hybrid was formed using cellulose fibers with LDH particles growing on their surface and then covered by abietic acid. The fibers were tested against hydrophobicity and lipophilicity.

Exfoliated nanosheets of layered calcium phenylphosphonate assisted by solvent were used in “Layered calcium phenylphosphonate: a hybrid material for a new generation of nanofillers” to promote the mechanical properties and improve the barrier effect for applications such as fire retardancy and gas permeation in a low molecular weight epoxy resin [28].

Regarding specific applications, the dielectric properties were investigated by broadband dielectric spectroscopy (BDS) in “Nanocomposite–parylene C thin films with high dielectric constant and low losses for future organic electronic devices” [29]. A combination of deposition techniques was used, chemical vapor deposition for parylene and RF-magnetron sputtering for silver nanoparticles. The content and size of the latter influences the dielectric characteristics of the resulting hybrid films. Such devices may find application as insulating gates in organic field-effect transistors (OFETs).

Optical properties are the focus in “Ceria/polymer nanocontainers for high-performance encapsulation of fluorophores” [30]. Here, an organic/inorganic system is based on a liquid core containing a fluorophore (terrylene diimide) within a polymer shell armored with an inorganic layer (cerium oxide nanoparticles). CeO_2_ nanoparticles act as oxygen scavengers, protecting the organic fluorophore from molecular oxygen. A different approach to luminescent composite films is reported in “Towards rare-earth-free white light-emitting diode devices based on the combination of dicyanomethylene and pyranine as organic dyes supported on zinc single-layered hydroxide” [31]. In this article, two fluorescent organic dyes, dicyanomethylene and pyranine, emit visible light upon blue LED excitation and are tethered to single layer hydroxide platelets and then embedded into a silicone polymer. These coatings deliver white-light emission when placed above a blue LED.

## Health

Functionalized nanoparticles are highly investigated as possible platforms for disease diagnosis and therapy, leading to potential applications in nanomedicine. The state-of-the-art, as well as potential further developments, are reviewed in “Targeting strategies for improving the efficacy of nanomedicine in oncology” [32]. Nanocarriers for drugs were also decorated with suitable moieties to tune their affinity with specific biological membranes. More sophisticated strategies, including double targeting, are also highlighted in several articles. Among others, nanoparticles are often used as specific agents in dual therapy and diagnostics (i.e., theranostics). In “Size-selected Fe_3_O_4_–Au hybrid nanoparticles for improved magnetism-based theranostics”, a Fe_3_O_4_–Au hybrid nanomaterial is simultaneously employed as a contrast agent in magnetic resonance imaging (MRI) and for local heating therapy using magnetic particle hyperthermia [33]. In vitro hyperthermia tests showed efficiency in inoculating mouse breast cancer cells. Another study reports the use of alendronate-coated gold nanoparticles [34]. The resulting gold–alendronate nanoplatform combines antitumor activity through drug delivery and photothermal therapy, as illustrated in vitro on the inhibition of prostate cancer cells.

In the field of hybrid coordination networks, new lanthanide-based networks synthesized by a solvo-ionothermal reaction or organic ligands are reported in “Magnetic and luminescent coordination networks based on imidazolium salts and lanthanides for sensitive ratiometric thermometry” [35]. Compounds associating the imidazolium ligand with several rare earth ions behave as a ratiometric thermometer and operate in the physiological range with suitable sensitivity.

Another metal–organic framework (MOF) is studied in “The nanoscaled metal–organic framework ICR-2 as a carrier of porphyrins for photodynamic therapy” [36]. Phosphinate-based MOF nanoparticles are decorated with porphyrin-type molecules as photosensitizers for biological applications. In this work, it was found that the photodynamic efficacy of the system depends on the substituent at the porphyrin phosphinate groups.

## Environmental

Hybrid nanomaterials may play a key role in the field of environmental research, in which environmental remediation and speciation can be targeted. For example, in the work “New micro/mesoporous nanocomposite material from low-cost sources for the efficient removal of aromatic and pathogenic pollutants from water”, a hybrid kaolinite nanocomposite was assembled via Zn cations upon calcination, resulting in a low-cost porous material exhibiting both micro- and mesopores [37]. The material is efficient in adsorbing water micropollutants, as well as the pathogen *E. coli.*, lending itself for application in water remediation. For the same application, a silica matrix, onto which the conjugated β-ketoenol–pyridine–furan ligand is immobilized, has been studied in “Removal of toxic heavy metals from river water samples using a porous silica surface modified with a new β-ketoenolic host” [38]. The metal adsorption speciation is relevant for some divalent cations in aqueous medium, and the hybrid system is recyclable.

Finally, catalysis is the focus of an article reporting the performance of functionalized gold clusters deposited on ZrO_2_ nanoparticles for benzyl alcohol oxidation in [39]. Interestingly, the defunctionalized gold nanoclusters exhibit full catalytic conversion.

Overall, this thematic issue clearly highlights not only the current trends in the field of hybrid materials but also a special focus on the comprehensive elaboration of new functional nanostructures. It also illustrates the consistently high potential of hybrid materials for numerous applications. We hope that readers will enjoy this reference work and find this thematic issue a source of inspiration for their own future research. It was an immense pleasure for us to edit this thematic issue of *Beilstein Journal of Nanotechnology* devoted to “Advanced hybrid nanomaterials”.

Verónica de Zea Bermudez, Fabrice Leroux, Pierre Rabu, Andréas Taubert

Vila Real, Aubière, Strasbourg, Potsdam, November 2019
